# Transcription Factors Pmr1 and Pmr2 Cooperatively Regulate Melanin Biosynthesis, Conidia Development and Secondary Metabolism in *Pestalotiopsis microspora*

**DOI:** 10.3390/jof8010038

**Published:** 2021-12-31

**Authors:** Mengkai Zhou, Ze Li, Yanjie Liu, Ping Zhang, Xiaoran Hao, Xudong Zhu

**Affiliations:** 1 Beijing Key Laboratory of Genetic Engineering Drug and Biotechnology, College of Life Sciences, Beijing Normal University, Beijing 100875, China; zhoumengkai0907@163.com (M.Z.); lize2020@mail.bnu.edu.cn (Z.L.); yunher@bnu.edu.cn (Y.L.); zp1516@bnu.edu.cn (P.Z.); 2 National Experimental Teaching Demonstrating Center, College of Life Sciences, Beijing Normal University, Beijing 100875, China; 2015xrhao@bnu.edu.cn

**Keywords:** transcription factor, melanin, conidia development, *Pestalotiopsis microspora*

## Abstract

Melanins are the common fungal pigment, which contribute to stress resistance and pathogenesis. However, few studies have explored the regulation mechanism of its synthesis in filamentous fungi. In this study, we identified two transcription factors, Pmr1 and Pmr2, in the filamentous fungus *Pestalotiopsis microspora*. Computational and phylogenetic analyses revealed that Pmr1 and Pmr2 were located in the gene cluster for melanin biosynthesis. The targeted deletion mutant strain *Δpmr1* displayed defects in biosynthesis of conidia pigment and morphological integrity. The deletion of *pmr2* resulted in reduced conidia pigment, but the mycelial morphology had little change. Moreover, *Δpmr2* produced decreased conidia. RT-qPCR data revealed that expression levels of genes in the melanin biosynthesis gene cluster were downregulated from the loss of Pmr1 and Pmr2. Interestingly, the yield of secondary metabolites in the mutant strains *Δpmr1* and *Δpmr2* increased, comparing with the wild type, and additionally, Pmr1 played a larger regulatory role in secondary metabolism. Taken together, our results revealed the crucial roles of the transcription factors Pmr1 and Pmr2 in melanin synthesis, asexual development and secondary metabolism in the filamentous fungus *P. microspora*.

## 1. Introduction

The production of pigments can be consistently observed during the growth and development of filamentous fungi, among which the most common forms are melanins. Melanins are polyphenol biopolymers that mainly distribute in the reproduction structure such as conidia in filamentous fungi [1]. Previous studies have shown that melanins contribute to high-radiation protection, microbe survival and fungal virulence [2,3]. For example, a large population of melanotic fungal species were isolated from the contaminated soil of Chernobyl [4], demonstrating the crucial roles of melanin in protecting against radiation exposure. Melanins also are critical for the virulence of pathogenic fungi and promote fungal invasion and colonization in humans, animals, and plants. In *Penicillium marneffei*, melanins block host defense mechanisms to increase fungal virulence [5]. Recent observations suggest that the production of melanin is also related to the integrity of fungal cell wall [6,7].

The enzymes that are responsible for melanin biosynthesis are usually polyketide synthase (PKS) in filamentous fungi [8]. PKS-encoding genes in fungi were frequently organized in gene clusters in which genes with related functions were grouped together and coregulated. Gene clusters for melanin biosynthesis have been characterized in several fungi [9,10]. These clusters mainly harbor genes encoding enzymes including PKSs, oxidases and reductases, as well as transcription factors (TFs). The transcription factors perform regulatory function in the synthesis of melanin and the growth and reproduction of fungi. In *Colletotrichum lagenarium*, the TF Cmr1 was required for pigment production and conidiogenesis [11]. Similarly, the transcription factor Amr1 in *Alternaria brassicicol* induced melanin biosynthesis and helped to UV tolerance [12]. Three pathway-specific TFs whose genes were clustered with *bcpks12* or *bcpks13* in *Botrytis cinerea*, and BcSMR1 regulated the expression of the adjacent pks gene responsible for sclerotial melanin production. BcZTF1 and BcZTF2 were dispensable for conidial melanogenesis [13]. In *Pestalotiopsis fici*, the melanin biosynthetic gene cluster (Pfma) included two TFs named PfmaF and PfmaH. PfmaH only regulated melanin biosynthesis, and PfmaF affected the production of melanin, secondary metabolism and fungal development [14].

*Pestalotiopsis microspora* NK17 was isolated by our laboratory, which can produce a number of secondary metabolites, such as dibenzodioxocinones, e.g., pestalotiollide B (an analog of a new class of cholesterol ester transfer protein inhibitor). Previously, we identified a polyketide synthase gene *pks1* for melanin biosynthesis in NK17 [15]. In this study, we further identified the melanin biosynthesis gene cluster and charactered two TFs, Pmr1 and Pmr2, in the cluster. The deletion of *pmr1* led to the loss of pigmentation in the conidia and changes in conidia morphology. Interestingly, we found that the yield of secondary metabolites in *Δpmr1* was significantly increased. Pmr2 was involved partially in melanin synthesis and secondary metabolism. In regulation, Pmr1 played a dominant over Pmr2, whereas Pmr2 was found to mediate spore development. The above results suggested Pmr1 and Pmr2 were critical regulators of pigment synthesis, conidia development and secondary metabolism in *P. microspora*.

## 2. Materials and Methods

### 2.1. Strains, Media and Growth Conditions

The fungus *P. microspora* NK17 was previously isolated by our laboratory [16]. *P. microspora* NK17 and its mutants were grown in potato lactose broth (PLB; 20% peeled and sliced potato, 2.0% lactose, natural pH) or on potato lactose agar (PLA; PLB with 2% agar) at 28 °C with shaking at 180 rpm. *Escherichia coli* strain DH5α and DB3.1 were cultivated in LB with appropriate antibiotics at 37 °C. *Agrobacterium tumefaciens* LBA4404 was grown in LB or inducing medium (IM) at 28 °C with shaking at 200 rpm. IM medium supplemented with acetosyringone (AS, Solarbio, Beijing, China) was used in the transformation of NK17.

### 2.2. Bioinformatic Analysis

Based on the gene sequence of the key enzyme PKS1, the melanin biosynthesis gene cluster was predicted using the software antiSMASH (https://antismash.secondarymetabolites.org, accessed on 29 October 2021) [17]. To further characterize the function of genes in the cluster, BlastP searching for orthologous genes (NCBI) was conducted. The amino acid sequences of the putative TFs were subjected to the NCBI Conserved Domains database to determine the conserved domains. A phylogenetic tree was built for the phylogenetic evaluation of Pmr1. Homologous protein sequences were acquired by protein blast searches. Then, the amino acid sequences of Pmr1 and selected homologues were aligned using MUSCLE algorithms in MEGA 11. The neighbor joining method was applied to construct the tree.

### 2.3. Gene Cloning, Plasmid Construction and Genetic Manipulation

The vectors for deletion were constructed by the OSCAR method as previously described [18]. Briefly, fragments homologous to the 5′ and 3′ franking regions of the target gene were obtained by PCR amplification and gel purified. Then, the purified fragments, pA-hyg-OSCAR, pOSCAR and BP clonase II enzyme (Invitrogen, CA, USA) were incubated at 25 °C overnight to construct the deletion vectors. 

Disruption of *pmr1* and *pmr2* in *P. microspora* was achieved through *Agrobacterium*-mediated fungal transformation. The deletion constructs were introduced into *A. tumefaciens* LBA4404. Then, the LBA4404 containing the vectors were cultured in IM medium with 40 mg/L acetosyringone at 28 °C. When OD_600_ reached to 0.5~0.8, the cells were co-cultured with 10^7^ conidia of NK17 at 28 °C on IM plate containing 40 mg/L AS on a nitrocellulose filter. After co-cultivation, the filter was transferred to the screening plate. After 48 h of incubation, the filter membranes were removed and transformant colonies were observed on the surface of the selection plates. 

To purify the transformants, single-spore isolation was conducted. Purified transformants were inoculated in PLB for total DNA extraction. PCR amplification was used for primary screening for correct transformants. In further confirmation, genomic DNA was extracted and subjected to Southern blotting. Genomic DNA of the transformant and the WT strains was digested with specific restriction endonucleases and fractionated on 0.7% agarose gel that was transferred onto a Magmaprobe Nylon Transfer Membrane-N^+^ (Osmonics, Minnetonka, MN, USA). DNA labeling, hybridization, and detection procedures were performed by following the protocol of the DIG High Prime DNA Labeling and Detection Starter Kit II (Roche China, Shanghai, China). An 869 bp DNA fragment that was generated by PCR amplification with the primer pair *pmr1*-up(F)/*pmr1*-up(R) was used to probe in Southern blots for *Δpmr1*, and a 553 bp probe generated with the primer pair *pmr2*-down(F)/*pmr2*-down(R) was used to verify the deletion of *pmr2* in the blots. The primers used in this experiment were listed in Supporting Information Table S1.

### 2.4. RNA Preparation and Quantitative Real-Time PCR

*P. microspora* NK17 and the right mutants grown in PLB for 6 days were used for total RNA extraction using the RNAiso Plus kit (Takara Bio, Shiga, Japan). The first strand of cDNA was synthesized and gDNA was removed following manufacture’s protocol of *TransScript*^®^ II One-Step gDNA Removal and cDNA Synthesis SuperMix (TransGen Biotech, Beijing, China). The qPCR protocol was performed in 20 μL reactions on a LightCycler^®^ 480 instrument III (Roche Diagnostics, Indianapolis, IN, USA). LightCycler^®^ 480 SYBR^®^ GreenⅠMaster Mix was used to perform three-step RT-PCR. The PCR cycle parameters were set as the previous description [19]. The $2^{-\Delta \Delta \mathrm{CT}}$ relative quantification method was used to calculate the relative expression levels of target genes. The gene encoding glyceraldehyde-3-phosphate dehydrogenase (GAPDH) was used as an internal reference. The primers used in this experiment were listed in Supporting Information Table S2.

### 2.5. Phenotype Observation

To observe colony morphology and pigment accumulation, NK17 and the confirmed mutants were inoculated on PLA at 28 °C, and the colony morphology and pigmentation were observed on daily basis. The strains also were grown in PLB with shaking at 200 rpm for 6 days, and were subject to filter and freeze-drying to determine the dry weight of mycelia.

Optical microscopy was used to observe the conidia production. Conidiation was achieved on PLA for 6 days. Conidia morphology and pigment were observed using 10×/0.25NA and 40×/0.65NA objective lenses on a Moticam Ⅹ3 microscope (Motic, Xiamen, China).

### 2.6. Conidia Quantification and Secondary Metabolite Profiling

Conidia were collected from 8-day-old cultures on PLA. The quantification for each strain was performed in triplicate. Each plate was washed three times with sterile distilled water and the conidia suspension were adjusted to appropriate volume. The concentration of conidia suspension was determined by hemocytometry under the Carl Zeiss Axio Imager Z2 Apotome2 Upright Microscope (Carl Zeiss, Oberkochen, Germany). 

To profile the secondary metabolites, equal numbers of conidia were cultured in 100 mL PLB, at 28 °C, 200 rpm for 6 days. The fermentation broth was subjected to vacuum filtration to remove the hyphae. 80 mL of ethyl acetate was added to the filtrate and the samples were placed at room temperature overnight to extract the secondary metabolisms. The extracted samples were re-dissolved in 1 mL methanol after concentration by rotary evaporation. All samples were filtered using 0.22 μm Millipore filters before loading. HPLC profiling was performed as previously reported [19].

## 3. Results

### 3.1. Chracterization of the Transcription Factors in the Gene Cluster of Melanin Biosynthesis 

**Figure 1:**
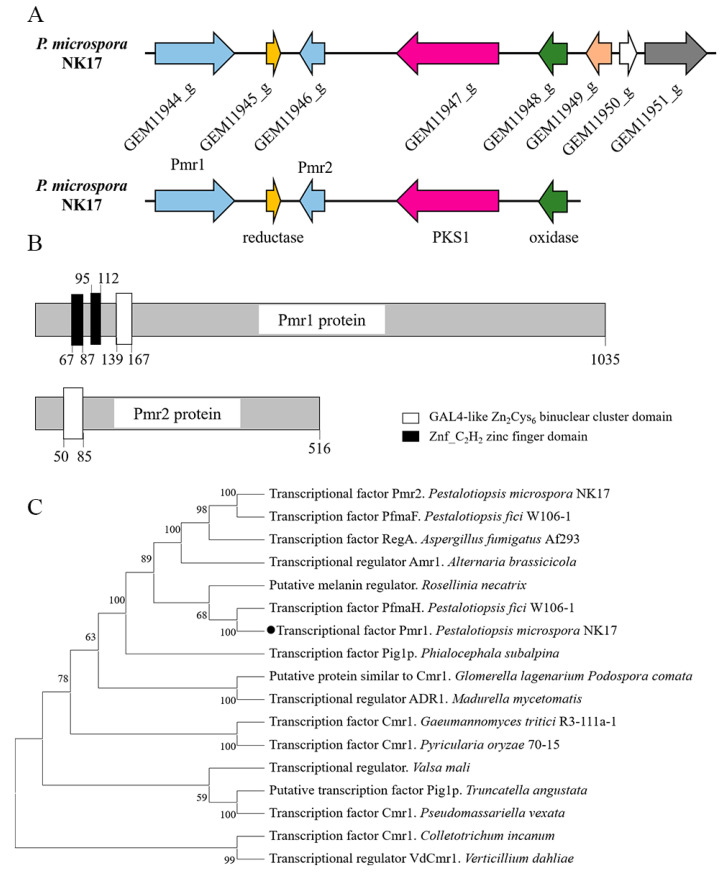
Structure of the *pks1* gene cluster for melanin biosynthesis and prediction of TFs in the cluster. (**A**) The *pks1* gene cluster includes eight genes, encoding oxidases, PKS, transcriptional factors and reductases that are remarkably conserved in *P. microspora* and other studied fungi. (**B**) Schematic of the domain architecture of TF Pmr1 and Pmr2. (**C**) Phylogenetic tree of Pmr1 was built with the neighbor-joining method by MEGA 11. Bootstrap supports >50% from 1000 replicates per run are labeled on branch nodes. TF Pmr1 of *P. microspora* is marked with a black dot.

In previous study, we demonstrated a polyketide synthase gene *pks1* responsible for melanin biosynthesis in *P. microspora* [15]. To further characterize the melanin pathway, we firstly identified the putative gene cluster with the antiSMASH software. The putative gene cluster included eight genes from GEM11944_g to GEM11951_g (Figure 1A). Furthermore, four genes of the cluster were found to be conserved for melanin synthesis between NK17 and other fungi. The conserved genes encode a putative multicopper oxidase (GEM11948_g), a putative 3-oxoacyl-(acyl-carrier-protein) reductase (GEM11945_g) and a putative transcription factor (GEM11944_g, Pmr1) in addition to the core enzyme PKS1. Notably, the homologous gene clusters in some studied fungi, like *A. fumigatus*, only contains one transcription factor [20]. In contrast, the predicted cluster in *P. microspora* contains two putative transcription factors, designated as Pmr1 (GEM11944_g) and Pmr2 (GEM11946_g).

Alignments against Conserved Domain Database suggested that Pmr1 contained two Znf_C_2_H_2_ zinc finger domains and a GAL4-like Zn_2_Cys_6_ binuclear cluster DNA-binding domain. Pmr2 only contained a GAL4-like Zn_2_Cys_6_ binuclear cluster domain (Figure 1B and Figure S1). Using the basic local alignment search tool (BLAST), we found that the amino acid sequence of Pmr1 was evolutionarily conserved (Figure S2). A phylogenetic tree built with the neighbor-joining method by the MEGA 11 showed that Pmr1 was a possible orthologue of Cmr1 (Figure 1C). However, no well-characterized counterparts (>40% identity) of Pmr2 was found in fungi. 

**Figure 2:**
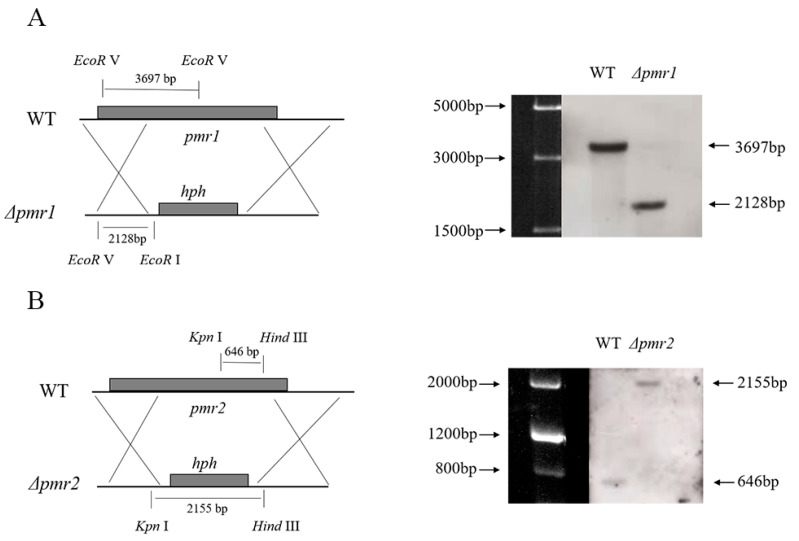
Confirmation of the disruption of *pmr1* and *pmr2.* (**A**) Southern blotting for verification of the *pmr1* deletion. The left panel shows the knock-out strategy for *pmr1* via homologous recombination and the restriction enzyme sites used in Southern blot are indicated. Genomic DNAs from NK17 and *Δpmr1* were digested with *EcoR* I and *EcoR* Ⅴ. One band of 2128 bp on the membrane was obtained for *Δpmr1*, and one band of 3697 bp was found for WT. (**B**) Southern blotting for verification of the deletion of *pmr2*. Genomic DNAs from NK17 and *Δpmr2* were digested with *Kpn* I and *Hind* Ⅲ. One band of 2155 bp on the membrane was obtained for *Δpmr2*, and one band of 642 bp was found in WT.

To investigate the function of the two putative TFs, we deleted their coding regions via homologous recombination utilizing a method of *Agrobacterium*-mediated T-DNA transformation as previously described [18]. The gene deletion cassette used *hyh* as selection maker. Eleven out of fourteen randomly picked hygromycin B resistant transformants showed obvious colony color changes in *pmr1* deletion. Nine out of fourteen randomly picked hygromycin B resistant transformants in *pmr2* showed a phenotype of conidia yield reduction. Diagnostic PCR and Southern blotting were used to verify positive transformants (Figures S3 and S4). In Southern blotting, 2128 bp band was detected as expected for the *pmr1* correct mutant and an anticipated 2155 bp band was detected for the *pmr2* mutant (Figure 2).

### 3.2. Effects of Pmr1 on Conidial Pigmentation and Morphogenesis 

**Figure 3:**
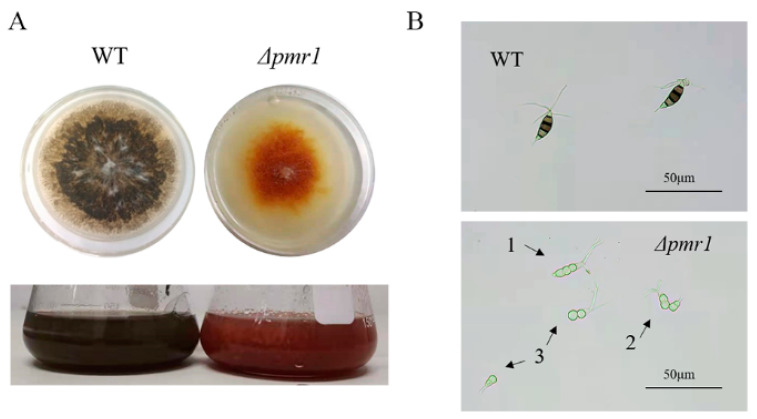
Pmr1 regulates melanin biosynthesis and conidial morphogenesis in *P. microspora*. (**A**) Phenotype of *P. microspora* WT and *Δpmr1* strains. Both strains were cultivated on PLA or in PLB for 7 days at 28 °C. The colonies of *Δpmr1* strain turned burgundy, and that of WT was in dark. (**B**) Morphology of conidia produced from WT and *Δpmr1* mutant. The original 5-celled fusiform conidia (upper panel) were deformed in *Δpmr1* (bottom panel).

To study the effect of Pmr1 on pigmentation, *P. microspora* WT and *Δpmr1* strains were grown on PLA plates at 28 °C for 7 days, and the colonies appeared orange in *Δpmr1* strains in comparison to the black color in WT (Figure 3A). When the mutants were grown in PLB liquid, the same phenotype was observed (Figure 3A). Since previous experiment demonstrated hyphae are colorless in NK17, the color of colonies mainly comes from conidia. Hence, color change in *Δpmr1* colony suggested a deficiency of conidial pigmentation. 

We observed the conidia using optical microscopy. Normal conidia in WT contain five cells, three of which were heavily pigmented, and the cell wall with a black pigment was observed. However, the conidia of *Δpmr1* mutants showed deficiency in pigment production both in the conidia and the cell wall. Simultaneously, a large number of deformed conidia were observed. Different from the five-celled conidia generated in WT, conidia of *Δpmr1* only contain two or three cells (Figure 3B). Further research revealed *Δpmr1* strains form five-celled, colorless conidia at the initial stage, then the initial five-celled conidia started to spall between the second and third cell to form two and three-celled conidia, respectively. The phenotype is consistent with that of the *pks1* deletion strains, which indicates the knockout of *pmr1* blocks melanin biosynthesis, and then affects conidial pigmentation and multicellular development. Due to the abnormal division of conidia in *Δpmr1*, it is difficult to accurately count the conidia quantity of the mutant; therefore, we could not determine the effect of Pmr1 deletion on conidia yield.

### 3.3. Pmr2 Influences Conidial Pigmentation and Conidia Production

**Figure 4:**
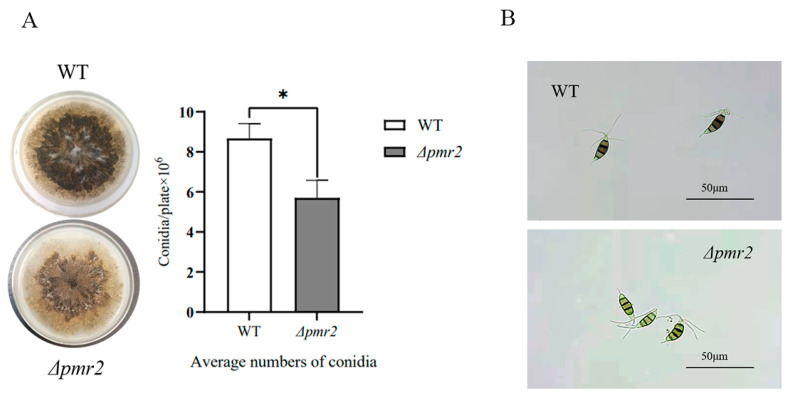
Effects of Pmr2 on melanin synthesis and conidia development. (**A**) Statistics of the number of conidia produced by WT and *Δpmr2*. The disruption of *pmr2* reduced the production of conidia. Three biological replicates were conducted for both strains. Error bars represent standard deviation. Asterisks indicate differences in mean values significantly (* *p* < 0.05). (**B**) Morphology of conidia of *Δpmr2*. Compared with the WT, the color of the conidia was lighter in *Δpmr2* strain.

To study the role of *pmr2* in conidiogenesis of NK17, we cultivated NK17 and *Δpmr2* strains on PLA at 28 °C for 7 days. As shown in Figure 4A, less conidia were observed on the plate of *Δpmr2*. After 7 days of cultivation on PLA, the mutant strain *Δpmr2* produced an average of 5.70 ± 0.88 × 10^6^ (*p* < 0.01) conidia per plate, whereas WT produced about 8.67 ± 0.74 × 10^6^ (*p* < 0.01) conidia per plate (Figure 4A).

When observing the conidia of *Δpmr2* with an optical microscope, we found that compared with WT, the conidia morphology was not much different, which was still a standard spindle-shaped, five-cell form. Notably, three median cells among the five cells were heavily pigmented with melanin in WT, whereas the pigmentation of the three median cells in *Δpmr2* was obviously less (Figure 4B). The results demonstrated that the deletion of TF Pmr2 also affected the synthesis of melanin, but to a lesser degree in comparison with the effect of Pmr1.

### 3.4. Roles of Pmr1 and Pmr2 in Biosynthesis of Secondary Metabolites

**Figure 5:**
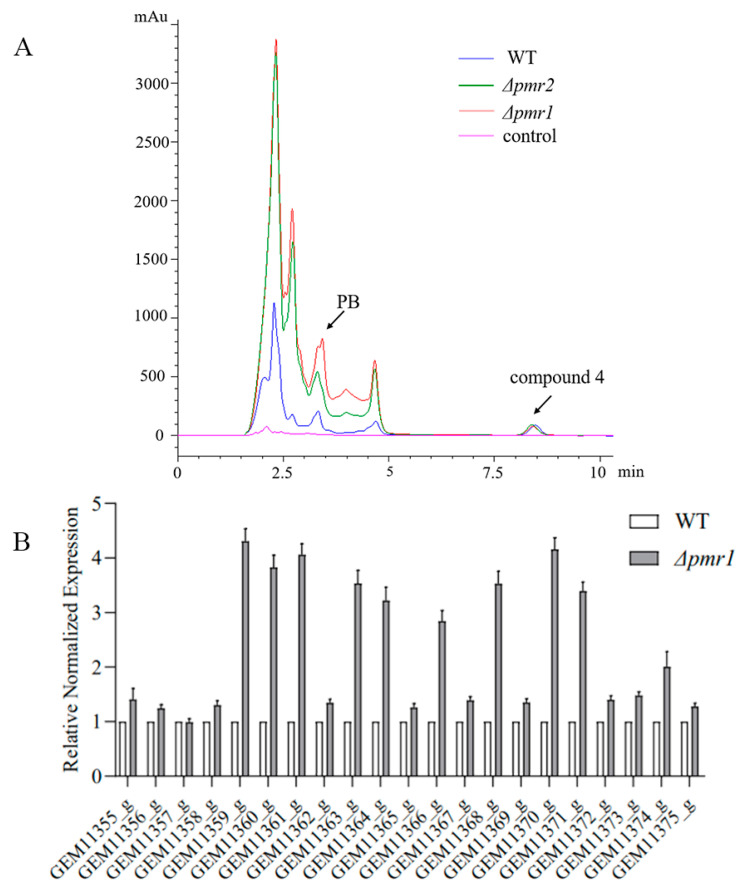

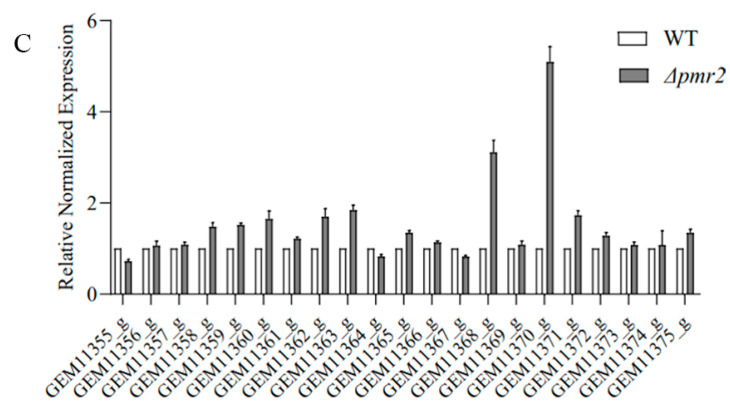
Pmr1 and Pmr2 negatively regulate the production of secondary metabolites. (**A**) HPLC analysis on the production of secondary metabolites in WT, *Δpmr1* and *Δpmr2* mutants. The analysis was repeated for three times. (**B**) RT-qPCR analysis of genes in dibenzodioxocinons biosynthesis gene cluster in *Δpmr1* strain grown in PLB for 8 days. The expression data were analyzed by the GraphPad 8. (**C**) RT-qPCR analysis on the expression levels of genes in dibenzodioxocinons biosynthesis gene cluster in *Δpmr2* strain cultivated in PLB for 8 days.

We explored the effect of Pmr1 and Pmr2 on secondary metabolites synthesis. The major secondary metabolites produced by NK17 are dibenzodioxocinins. Among them, pestalotiollide B (PB) is the most rebundant one. Previously we characterized a gene cluster harboring 21 genes (from GEM11355_g to GEM11375_g), responsible for regulating the production of dibenzodioxocinins [21]. In order to probe the influence of Pmr1 and Pmr2 on secondary metabolites synthesis, we measured the expression of genes in the cluster using RT-qPCR in *Δpmr1*, *Δpmr2* and WT strains. RT-qPCR analysis showed that expression of most genes in the cluster was significantly upregulated except GEM11357_g whose expression level was little changed in the mutant strain *Δpmr1* (Figure 5B). Similarly, except for GEM11355_g, GEM11364_g and GEM11367_g, other genes responsible for the synthesis of dibenzodioxocinins were upregulated due to the deletion of *pmr2* (Figure 5C).

Then, we conducted HPLC profiling for these mutant and wild-type strains. Previous experiments showed that production of secondary metabolites reaches its peak at 8 days, an 8-day culture was used for the extraction of secondary metabolites. HPLC analysis of metabolites revealed that the pestalotiollide B peak at 3.3 min was much greater in the *Δpmr1* and *Δpmr2* strains than that in the WT; moreover, this increment in *Δpmr1* was significantly greater than in *Δpmr2*. Interestingly, compound 4, a typical compound with unknown structure in *P. microspora*, was detected at a similar level in both WT and the mutant strains (Figure 5A). 

### 3.5. Pmr1 and Pmr2 Both Regulate the Expression of Genes in Melanin Biosynthesis Cluster

**Figure 6:**
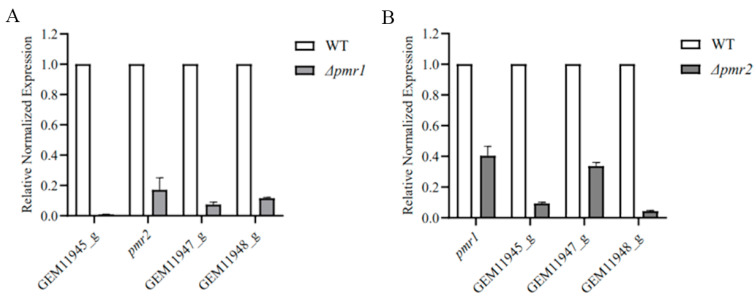
Pmr1 and Pmr2 positively regulates the expression of genes related to melanin synthesis. (**A**) RT-qPCR analysis of other genes in melanin synthetic gene cluster in *Δpmr1* strain grown in PLB for 7 days. The expression data were analyzed by the GraphPad 8. The gene expression quantity of wild type was standardization to ‘1’. (**B**) RT-qPCR studies on transcription levels of other genes in melanin synthetic gene cluster in *Δpmr2* strain grown in PLB for 7 days. The expression data were analyzed by the GraphPad 8.

As presented above, we identified five genes in the gene cluster for melanin synthesis, which encode a polyketide synthase, a putative multicopper oxidase, a putative 3-oxoacyl-(acyl-carrier-protein) reductase and two transcription factors, Pmr1 and Pmr2. To investigate the potential regulatory roles of Pmr1 and Pmr2 on the other genes in the cluster, we determined the expression of these genes using RT-qPCR. The results showed that Pmr1 or Pmr2 deletion downregulated the expression of the genes in melanin biosynthesis cluster, and the regulatory action of Pmr1 appeared significantly greater than the action of Pmr2 (Figure 6). 

This qPCR result was consistent with the previous microscopic observation (Figure 3B and Figure 4B). *Δpmr1* displayed a lack of melanin in conidia, whereas *Δpmr2* still had melanin in conidia, though showed a weaker accumulation. These results demonstrate that both Pmr1 and Pmr2 affect melanin biosynthesis by regulating the expression of related synthases in melanin biosynthesis cluster. In this process, Pmr1 plays a major regulatory role, and Pmr2 has a secondary role.

## 4. Discussion

Many fungi produce melanin, a widespread biologically protective pigment. It is multifunctional, not only essential for fungal resistance to environmental stresses, but also contributes to conidia development. The biosynthesis of melanin is catalyzed by a variety of enzymes, including oxidase, reductase and the core enzyme-polyketide synthase [22,23]. In addition, melanogenesis is also regulated by a variety of TFs. In this study, we identified two TFs, Pmr1 and Pmr2, which play a key role in melanogenesis in *P. microspora*. We found that the two TFs could not only promote the melanin synthesis, but mediate spore development and secondary metabolism as well. 

Previously, we identified the gene encoding PKS1, which is the core enzyme of melanin synthesis in NK17 [15]. On this basis, we predicted the gene cluster for melanin synthesis where *pks1* was located and analyzed the functions of other genes in the cluster by bioinformatics analysis. Additionally, two putative TFs, Pmr1 and Pmr2, were charactered. 

We demonstrated that the disruption of Pmr1 led to a significant decrease in the pigmentation of the colony, which became burgundy that different from the dark color of the WT. This is due to a melanin deficient phenotype of the conidia of *Δpmr1*. The result demonstrated that Pmr1 is critical for melanin production in conidia. Meanwhile, we found that the deletion of Pmr2 also affected the synthesis of melanin. It is worth noting the regulation action of Pmr2 contributed a secondary part to melanization of conidia of the fungus. The conidia of *Δpmr2* were still black, but had a lighter color than the WT (Figure 4B). Similar phenotype was also observed in other fungi [24,25]. Taken together, these results demonstrated Pmr1 and Pmr2 in our fungus coordinately regulate melanin synthesis, and Pmr1 plays a major role in this process.

Interestingly, we found a significant alteration in the morphology of conidia in *Δpmr1*. The wild-type conidia were fusiform and characterized by 5-celled form with three deeply pigmented central cells and hyaline terminal cells, three setulae (apical appendage) and a basal appendage were arising from the apex. However, the conidia of *Δpmr1* were no longer in the typical fusiform, and fractures occurred in the one end of the multicellular conidia, forming deformed conidia with two or three cells (Figure 3B). In *P. fici* which also belongs to the form genus *Pestalotiopsis,* TF PfamH was found to affect conidia morphology by regulating the production of melanin [14]. The discovery of essentiality of melanin for conidia morphology is common in filamentous fungi and is not limited to the *Pestalotiopsis* genus [26]. The deletion of *pmr2* also affected melanin synthesis but did not cause abnormal conidia morphology. 

Notably, in addition to regulating melanin biosynthesis and conidia mophogenesis, Pmr1 and Pmr2 also influence the production of secondary metabolites. NK17 produces a variety of secondary metabolites, most of which belong to the class of dibenzodioxocinones. Previous studies have pointed out that the lack of melanin will influence the production of other secondary metabolites, for example, the synthesis of chaetoglobosin A in *Chaetomium globosum* and the production of T-toxin in *Cochliobolus heterostrophus* [27,28]. In *P. fici*, the deletion of TFs Pfam H and Pfam F did not affect the secondary metabolism, but the overexpression of *Pfam F* increased the production of most secondary metabolites [11]. In this study, we found that the deletion of *pmr1* and *pmr2* both increased the production of other secondary metabolites, and Pmr1 had a more significant regulatory effect in this case. RT-qPCR analysis also showed that most genes in the dibenzodioxocinins biosynthesis gene cluster were simultaneously upregulated. These results are divergent to the previous observation of *P. fici*. Our results revealed that Pmr1 and Pmr2 in *P*. *microsopra* both have key roles in the biosynthesis of secondary metabolites in NK17. Considering their regulatory roles in melanin synthesis, we speculate that they may not directly act on the synthesis of secondary metabolites, but act like through the effects on the synthesis of melanin, since melanin biosynthesis and secondary metabolite production may compete for common precursors in upstream steps. Thus, inhibition of melanin synthesis may provide surplus precursors to secondary metabolite synthesis, thus promote their yield. More studies need to be carried out to prove this hypothesis.

In conclusion, our work shows that Pmr1 and Pmr2 in the melanin synthesis gene cluster coordinate melanin production, and their functions are essential for conidia development and secondary metabolism. Different from the previous single transcription factor regulation mechanism, we first discovered a dual mode of transcription factor regulation for melanin synthesis, conidia development and secondary metabolism in fungi. It is worth noting that Pmr1 is the major regulator in these processes, while Pmr2 has a secondary regulatory part in all these processes. The finding suggests that TFs can function as gene cluster specific regulators, and can also participate in the regulation of multiple pathways. This study provides information for the improvement of secondary metabolite biosynthesis.

## Data Availability

All relevant data are available in the Supplementary Materials.

## Supplementary Materials

The following supporting information can be downloaded at: https://www.mdpi.com/article/10.3390/jof8010038/s1, Figure S1: The predicted functional domains of Pmr1 and Pmr2. Figure S2: Amino-acid sequence (complete sequence) alignments of Pmr1. Figure S3: Diagnostic PCR screening for *pmr1* deletion mutants. Figure S4: Diagnostic PCR screening for *pmr2* deletion mutants. Table S1: Primers used for gene knockout and validation. Table S2: Primers used in quantitative real-time PCR.
