# Supplementary material for: Transcription Factors Pmr1 and Pmr2 Cooperatively Regulate Melanin Biosynthesis, Conidia Development and Secondary Metabolism in Pestalotiopsis microspora
Source: J Fungi (Basel). 2021 Dec 31;8(1):38. doi: 10.3390/jof8010038 (PMC8781371; doi:10.3390/jof8010038)
Supplement: Supplementary file 1 [file jof-08-00038-s001.zip › jof-1522742-supplementary.pdf]

Journal of Fungi

Supplementary Materials

Transcription factors Pmr1 and Pmr2 cooperatively regulate melanin biosynthesis, conidia development and secondary metabolism in *Pestalotiopsis microspora*

Mengkai Zhou [1], Ze Li [1], Yanjie Liu [1], Ping Zhang [1], Xiaoran Hao [2] and Xudong Zhu [1,*]

[1] Beijing Key Laboratory of Genetic Engineering Drug and Biotechnology, College of Life Sciences, Beijing Normal University, Beijing 100875, China

[2] National Experimental Teaching Demonstrating Center, College of Life Sciences, Beijing Normal Universi-ty, Beijing 100875, China

*** Correspondence**:

zhu11187@bnu.edu.cn (X.D. Zhu)

Tel: +86-010-58804266

ASSOCIATED CONTENT

Supplementary Tables and Figures

Table S1. Primers used for gene knockout and validation.

Table S2. Primers used in quantitative real-time PCR.

Figure S1. The predicted functional domains of Pmr1 and Pmr2.

Figure S2. Amino-acid sequence (complete sequence) alignments of Pmr1.

Figure S3. Diagnostic PCR screening for *pmr1*deletion mutants.

Figure S4. Diagnostic PCR screening for *pmr2* deletion mutants.

Table S1. Primers used for gene knockout and validation

**Primer name**	**Sequence (5′-3′)**
Pmr1-UF	GGGGACAGCTTTCTTGTACAAAGTGGAACCGTGTAATCGTTGTGCT
Pmr1-UR	GGGGACTGCTTTTTTGTACAAACTTGTTCTCGGCTTGTTCCTCAT
Pmr1-DF	GGGGACAACTTTGTATAGAAAAGTTGTTTGCCAGATGCTGAAACATGCT
Pmr1-DR	GGGGACAACTTTGTATAATAAAGTTGTGCGTGACAGGAAAGAAAAGACC
Pmr1-KO-V-F	GAAACGCTGCCAACAAACA
Pmr1-KO-V-R	TCCAAGCCCTGGGGACTAT
Pmr2-UF	GGGGACAGCTTTCTTGTACAAAGTGGAACAACAGCAGCGGTCATCT
Pmr2-UR	GGGGACTGCTTTTTTGTACAAACTTGTCATCTCACCAGCCTCCAT
Pmr2-DF	GGGGACAACTTTGTATAGAAAAGTTGTTGTCACCCTCTTGTCTTCCTC
Pmr2-DR	GGGGACAACTTTGTATAATAAAGTTGTATCAACTGCCGCATCTCG
Pmr2-KO-V-F	TCCACCAAGACTTGACATCG
Pmr2-KO-V-R	AGACCAGAAGGATAGAGGAGAC

* The underlined parts indicate the homologous arms of the primers for BP reaction.

Table S2. Primers used in quantitative real-time PCR

Primer name	Sequence (5′-3′)
qPmr1-F	GCATAAGCAGCGGGAGTC
qPmr1-R	TGGTTGGTCGGGAATGAA
q11945-F	GCAGCCAACAACAGCAAA
q11945-R	GGCAAGTTCAGCCACGAC
qPmr2-F	CCAACAGCAGCGGTCAT
qPmr2-R	GAGCGGTGGGCGAATAT
qPks1-F	GCCATAGGGAATAACGAGAA
qPks1-R	AGAGACAGAGACCAAAGCCC
q11948-F	AGAAGACGCCGACCTTGTAT
q11948-R	CGACCACCTGGAACTGATGT
qGAPDH-F	CGCATTGGTCGTATCGT
qGAPDH-R	ACCGTGGGTGGAGTCGT
q11355-F	CGATGACAAGGCATTAAGC
q11355-R	GAGCATAGTCGGCGTAGA
q11356-F	TGTGCAACTGGGAACACT
q11356-R	TTGGATGAGACACTGCCTAC
q11357-F	TTCACAGGAGACAACATACT
q11357-R	CTAACCTTACTCGCCAGTT
q11358-F	ACCACCTCGTTCACAATC
q11358-R	CGCTGAAGAAGTCATCGT
q11359-F	GTCGCAGCACTATCTCAC
q11359-R	GGAACTGGACGAACTTGG
q11360-F	ATATTCCGTCTTGTCAGTCTC
q11360-R	CGAGTAATGTTGGCTGTGA
q11361-F	CGGTATCGAGACATTGACA
q11361-R	CTGCTTGGCGTAGTTGAT
q11362-F	GTCAACAAGTCTGCGTATG
q11362-R	GTGCGAATATGCTGGAGA
q11363-F	TCGCCGTCACGATTAGTT
q11363-R	TCTCCATTTGGCTGCTTT
q11364-F	CACCGTCCGTGTCAGTA
q11364-R	TCCGCAGTAACAGCATC
q11365-F	TTGGACTATGAGACGGGAGA
q11365-R	GTGGAGATGGATCGGGTG
q11366-F	GCCGATGTTGGATGTGAG
q11366-R	CGTGCTTGGGTCCCTGAT
q11367-F	GAGGATGACTGGCTTACC
q11367-R	ATTGACGACACCGACATT
q11368-F	TGGATTGGTGAATGGATGG
q11368-R	TTGAGACTGTTGTGGATGG
q11369-F	GGTGTATGGATAGGCTTCAA

q11369-R	CAGCAATCAGCACTTCCT
q11370-F	CTGCCAACCTCCACTATAC
q11370-R	CCAGAGTCCTTGTCCAATT
q11371-F	AACTATCGGCGGCTTCTT
q11371-R	GTGGCAGAGATGGTGGTA
q11372-F	GTCTACGATTGGGCTGAG
q11372-R	CCGATGAAGTGCTGAGTC
q11373-F	CGACTGTGGCAAGAAGAA
q11373-R	TTACGGACGAGAGGTGAA
q11374-F	GCCAGAAGTTGTTGTAATACC
q11374-R	TGTCTAACGGATTCGCAATA
q11375-F	TTGGACTGTTCCGTATATGG
q11375-R	CTGGATGTTGACCGATAGG

Pmr1

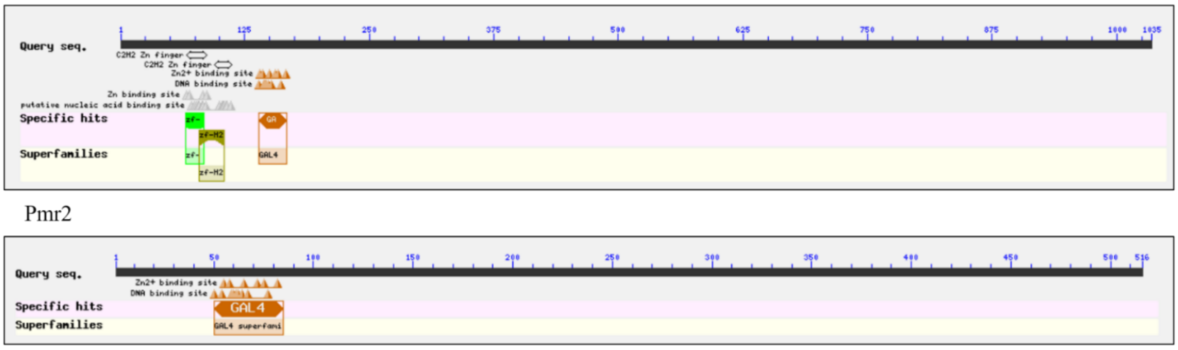

Figure S1. The predicted functional domains of Pmr1 and Pmr2. Pmr1 contained two Znf_C_2H_2 zinc finger domains and a GAL4-like Zn_2Cys_6 binuclear cluster DNA-binding domain. Pmr2 only contained a GAL4-like Zn_2Cys_6 binuclear cluster domain.

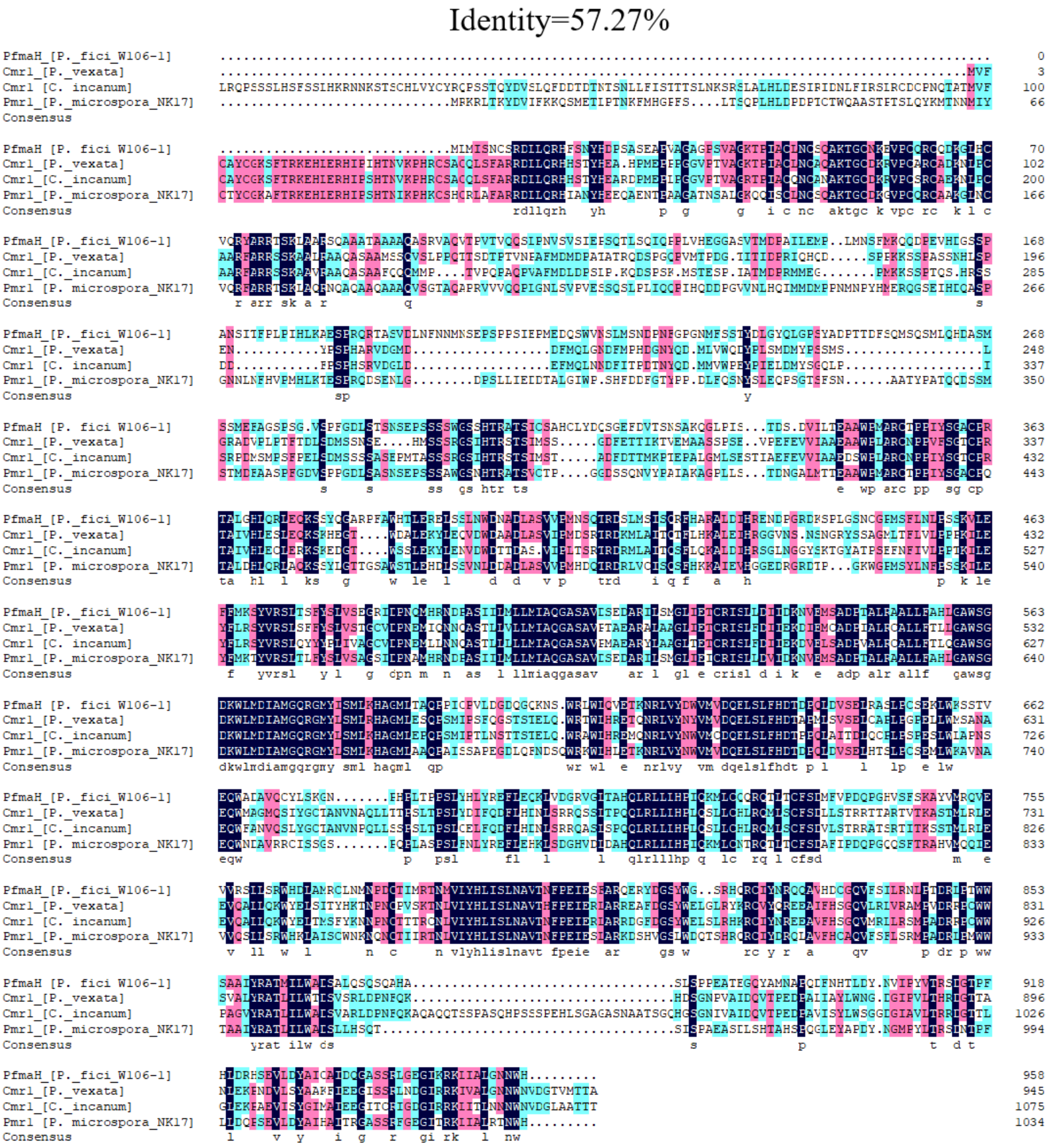

Figure S2. Amino-acid sequence (complete sequence) alignments of Pmr1 homologs from Pest*alotiopsis fici* W106-1, *Pseudomassariella vexata* and *Colletotrichum incanum*. Numbers indicated the length and the overall percentage of amino acid sequence identity was shown on the top. The identical amino acids were highlighted in blue.

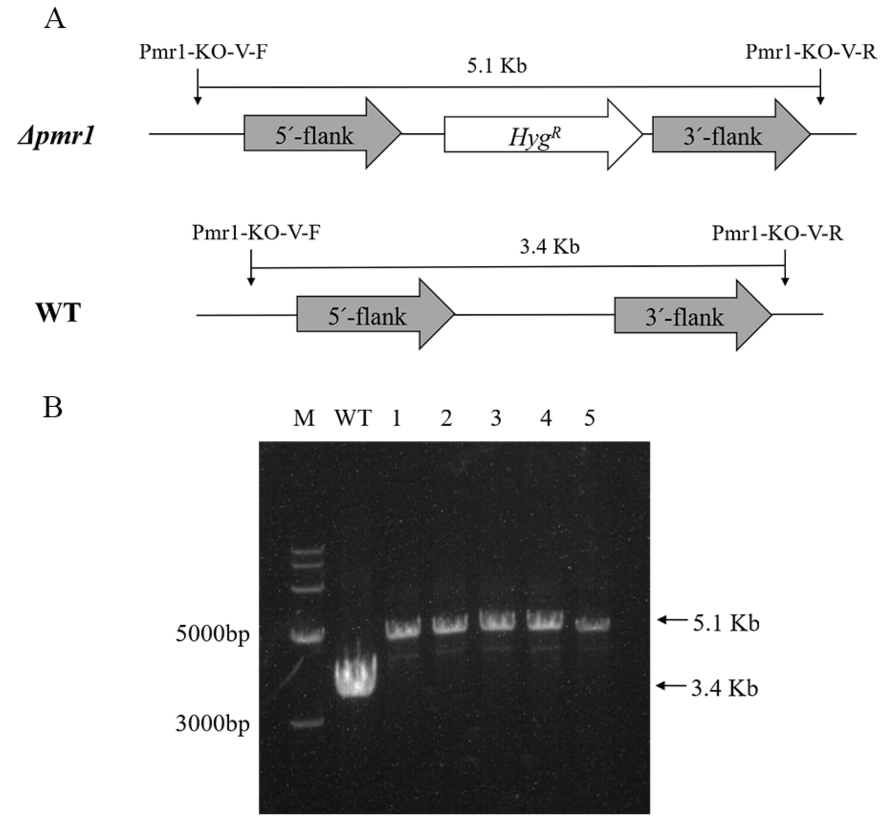

Figure S3. Diagnostic PCR screening for *pmr1* deletion mutants. (**A**) Schematic illustration of diagnostic PCR of *Δpmr1*. Primers pairs Pmr1-KO-V-F/Pmr1-KO-V-R were used for transformant screening. (**B**) Confirmation of *Δpmr1* strains by diagnostic PCR. A 5.1 Kb fragment could be amplified by the primers pair in mutants, while a 3.4 Kb fragment could be amplified in WT.

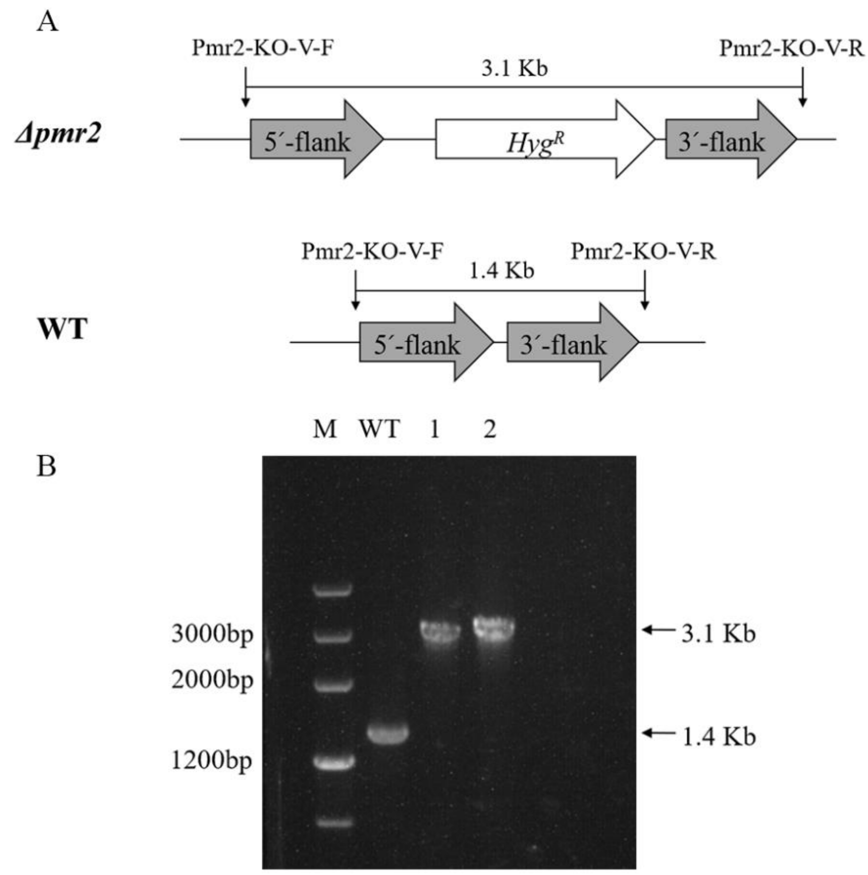

Figure S4. Diagnostic PCR screening for *pmr2* deletion mutants. (**A**) Schematic illustration of diagnostic PCR of *Δpmr2*. Primers pairs Pmr2-KO-V-F/ Pmr2-KO-V-R were used for transformant screening. (**B**) Confirmation of *Δpmr2* strains by diagnostic PCR. A 3.1 Kb band was amplified by the verify primers pair in *Δpmr2* mutants, while a 1.4 Kb band was amplified in WT. M: Maker Ⅲ DNA Marker.
